# Supplementary material for: Comprehensive Annotation and Functional Exploration of MicroRNAs in Lettuce
Source: Front Plant Sci. 2021 Dec 24;12:781836. doi: 10.3389/fpls.2021.781836 (PMC8739914; doi:10.3389/fpls.2021.781836)
Supplement: Supplementary file 3 [file Data_Sheet_9.ZIP › GSM2230753.plot/Lsa-miR167a_Lsat_1_v5_gn_4_9441.1_468_TPlot.pdf]

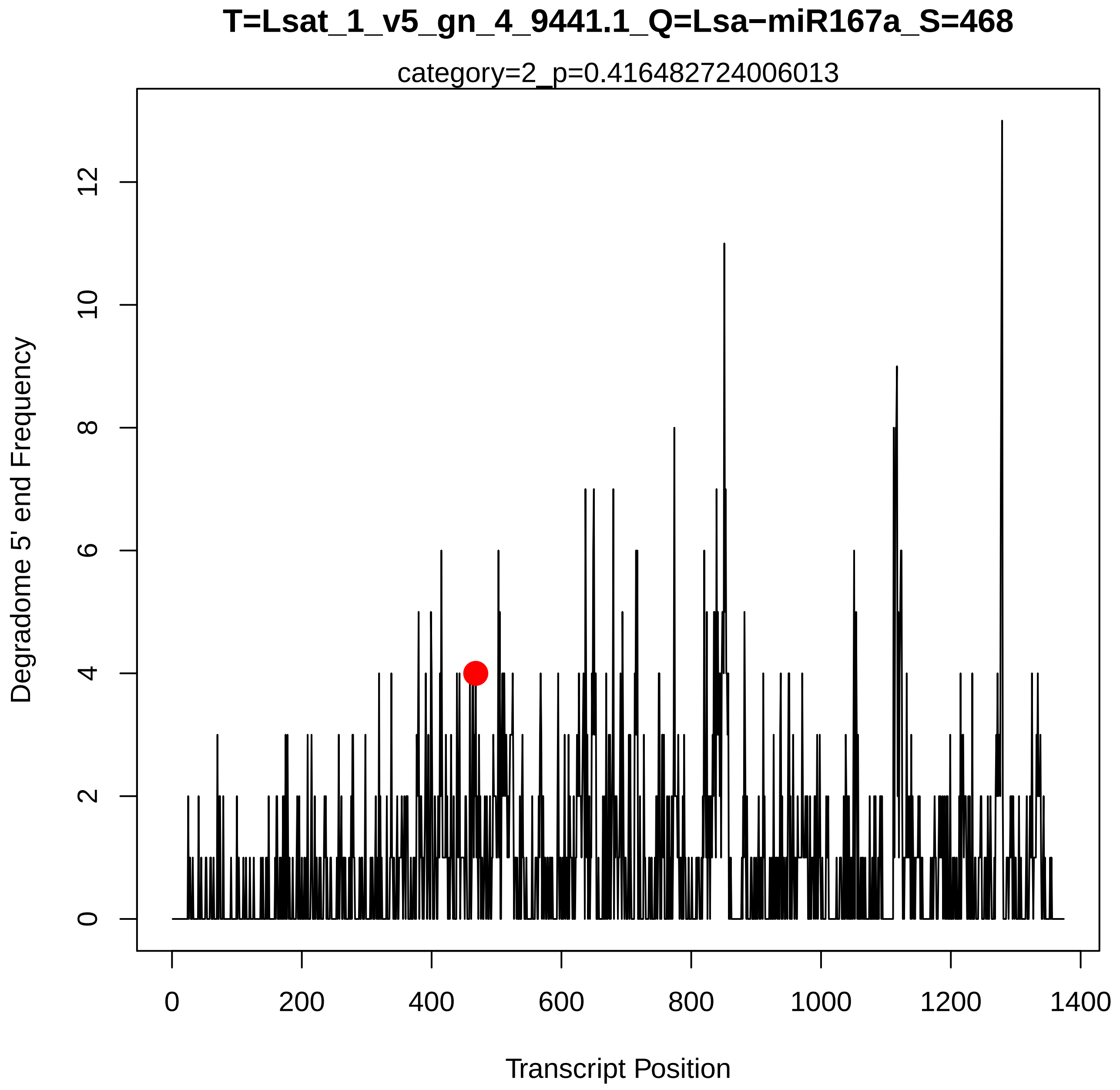

T=Lsat_1_v5_gn_4_9441.1_Q=Lsa−miR167a_S=468
category=2_p=0.416482724006013
Degradome 5' end Frequency
0
2
4
6
8
10
12
0
200
400
600
800
1000
1200
1400
Transcript Position
